# Design, Synthesis, Anticonvulsant Evaluation, and Molecular Docking Studies of New GABA_A_ Agonist Derivatives

**DOI:** 10.5812/ijpr-157392

**Published:** 2025-03-02

**Authors:** Mansur Nassiri Koopaei, Parsa Moghimirad, Ebrahim Saeedian Moghadam, Nasrin Nassiri Koopaei, Massoud Amanlou, Tahmineh Akbarzadeh, Mohammad Sharifzadeh, Mohsen Amini

**Affiliations:** 1 Department of Medicinal Chemistry, Faculty of Pharmacy, Tehran University of Medical Sciences, Tehran, Iran; 2 Drug Design and Development Research Center, The Institute of Pharmaceutical Sciences (TIPS), Tehran University of Medical Sciences, Tehran, Iran; 3 Department of Pharmaceutics, Faculty of Pharmacy, Tehran University of Medical Sciences, Tehran, Iran; 4 Department of Pharmacology, Faculty of Pharmacy, Tehran University of Medical Sciences, Tehran, Iran

**Keywords:** GABA_A_ Agonist, Molecular Docking, Synthesis, Anticonvulsant

## Abstract

**Background:**

The side effects and drug resistance associated with current antiepileptic drugs necessitate the design and synthesis of new candidate anticonvulsant agents.

**Objectives:**

The present study aimed to design, synthesize, and screen a new series of gamma-aminobutyric acid (GABA) agonist derivatives for the treatment of seizures in an animal model.

**Methods:**

The test chemical compounds were synthesized using known synthetic routes, and their structures were confirmed by various spectroscopic methods. Anticonvulsant activity was evaluated using the pentylenetetrazole (PTZ) animal model. Molecular docking studies were conducted to assess interactions with the GABA_A_ receptor.

**Results:**

Some synthesized compounds significantly improved seizure symptoms and reduced mortality rates in the PTZ model. Derivative 3c demonstrated a correlation with the GABA_A_ receptor in the flumazenil test.

**Conclusions:**

The synthesized molecules exhibited moderate to good activity compared to the control group. Derivative 3c notably increased seizure latency relative to the control. Flumazenil inhibitory effect tests indicated that 3c protects against PTZ-induced seizures via the synaptic GABA_A_ receptor.

## 1. Background

Epilepsy is the fourth most common neurological disease in the world. Approximately 0.5% to 1% of the world's population suffers from epilepsy (1). Seizures result from abnormal electrical activity in the brain, and in most cases, treatment begins with an antiepileptic drug. About half of the patients are controlled with single-drug therapy, and about 30% of patients need to take two or three drugs to control seizures. The remaining patients, who are resistant to oral antiepileptic drugs, require other treatments, including surgery (2-4). Excessive neuronal excitability leads to the formation of an epileptic center in specific regions of the brain. Convulsive attacks originate from the epileptic center. In epilepsy, both the hyperexcitability of neurons and the reduction of activity of GABAergic neurons are important. The GABA is an important neurotransmitter in the central nervous system (CNS) that inhibits the excitation of neurons or reduces their activities. Gamma-aminobutyric acid (GABA) receptors are divided into two types, GABA_A_ and GABA_B_ (5-7). The ion channel for transmission of the GABA effect distinguishes the GABA_A_ receptor, which is one of two ligand-gated ion channels. Stimulation of this receptor reduces the excitability of neurons by increasing the flow of negative chloride ions into the cell (8, 9).

**Figure 1.**
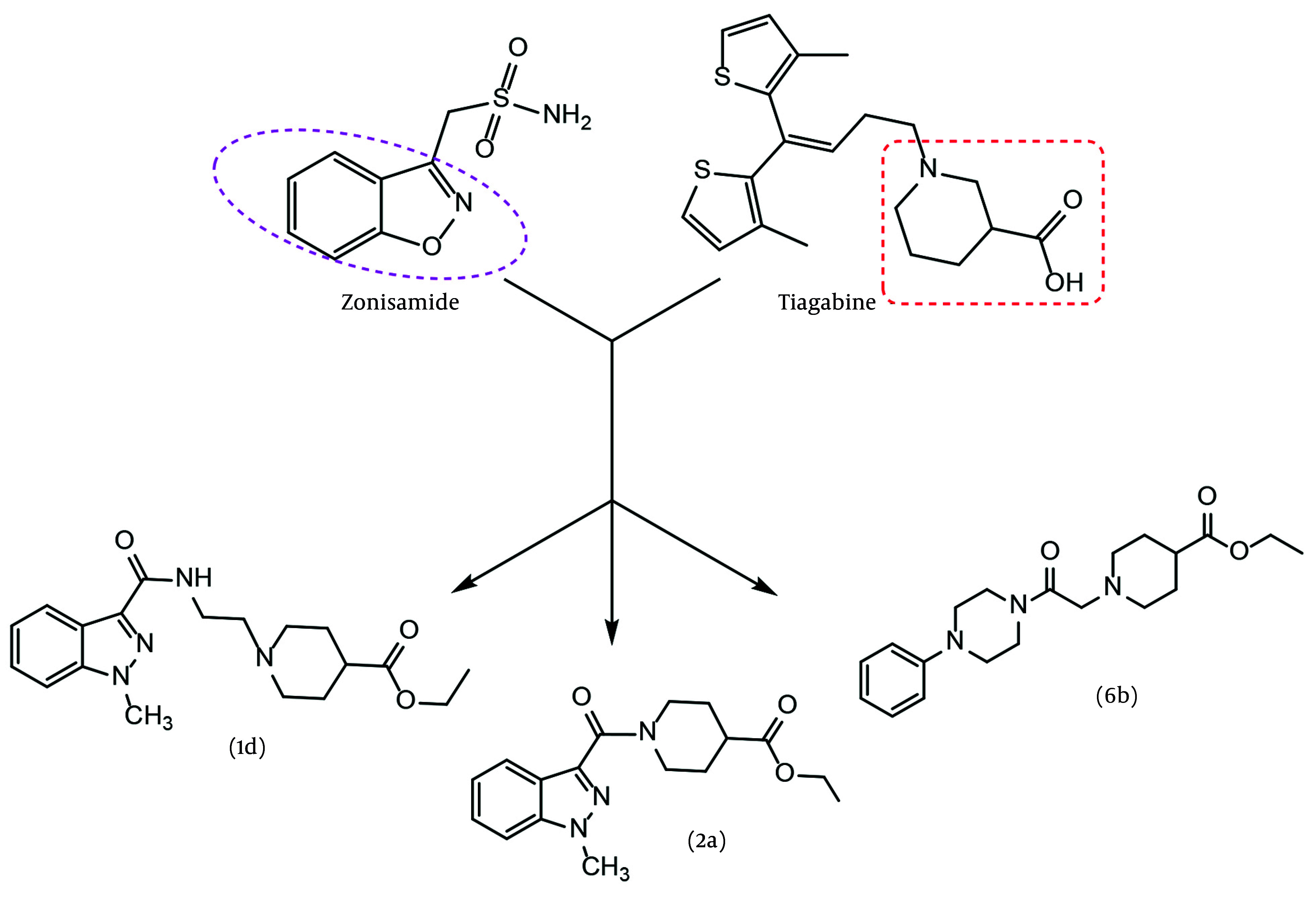
Design hybrid of new gamma-aminobutyric acid (GABA) agonist derivatives 1d, 2a and 6b

Studies on the anticonvulsant effects of various compounds and drugs have consistently attracted researchers. Two primary goals can be identified for these studies: (1) Discovering new and more effective drugs for the treatment and control of epilepsy, and (2) gaining a better and deeper understanding of the mechanisms of this disease and its accompanying phenomena (9). Our design is based on a new hybrid of two pharmacophores adapted from two approved antiepileptic drugs, zonisamide and tiagabine (Figure 1). Zonisamide is approved for adjunctive and monotherapy for seizures and atypical absence in adults. In Australia, it is marketed as both an adjunctive and monotherapy for partial seizures only (10). Tiagabine is a potent and selective inhibitor of the GABA transporter in the CNS. In both structures, two main pharmacophores can be identified: An aryl group positioned at a suitable distance from an anion or negative part moiety. However, in the case of tiagabine, two identical aryl groups (methyl thiophene) can be observed in a similar position. This specific arrangement of the second aryl group allows it to mimic the role of the first one. Inspired by the efficient pharmacophores of two recent antiseizure agents, a new series of GABA_A_ agonist derivatives, incorporating a hybrid of two pharmacophores, were designed, synthesized, and evaluated as antiseizure agents. In our design, three aryl groups — indazole, 4-chlorophenyl, and phenyl — were selected as the aromatic core. Several linker groups were used to connect the aryl core to the anion part of the molecules. The criteria for selecting the spacer included synthesis capability, chemical stability, and previous use in active structures. Additionally, an ester group, as a precursor of carboxylic acid, was embedded as the negative part of the molecule, except for 3c. The partial negative charge of 3c was provided by an amide bond. A variety of linker choices were considered to provide varying distances between the negative part of the molecule and the aromatic core.

## 2. Objectives

In the present study, the design and synthesis of a new series of GABA_A_ agonist derivatives are presented. Docking studies and evaluations of biological activity were performed. The mechanism of action was studied through in vivo experiments.

## 3. Methods

### 3.1. Chemistry

Laboratory-grade solvents, pentylenetetrazol (PTZ), potassium carbonate, and all reagents were purchased from Merck (Merck, Germany). 1-Methyl-1H-indazole-3-carbonyl chloride (CAS No: 106649-02-9) was obtained from Pharmaffilates (Parchkula, India). A Bruker 500 MHz Ultra spectrometer (Bruker, Darmstadt, Germany) was used for recording HNMR and CNMR spectra at 500 MHz and 125 MHz, respectively, using tetramethylsilane (TMS) as an internal standard. The symbols s (singlet), d (doublet), t (triplet), and m (multiplet) were used for spin multiplicities, and the J values are illustrated in Hertz (Hz). The IR data were obtained using a Nicolet FTIR 550 (Magna) instrument (Nicolet Instrument Corporation, Madison, WI, USA) with KBr disks. Melting points were measured with an Electrothermal IA9200 digital programmable melting point apparatus. The progress of reactions and evaluation of the purity of the compounds were checked with TLC. The partition coefficient values (log P) of the synthesized compounds were predicted using ChemDraw Ultra software version 12.0.

#### 3.1.1. General Procedure for the Synthesis of Compounds 1c, 1d, 2a, 3c, 4b, 5, and 6b

##### 3.1.1.1. 2-(1-Methyl-1H-indazol-3-yl)-4,5-dihydrooxazole (1c)

To a solution of 1-methyl-1H-indazole-3-carbonyl chloride 1a (2 mmol) in acetonitrile (20 mL), bromoethylamine (2.1 mmol) and potassium carbonate (2.5 mmol) were added. The mixture was stirred for 5 hours under reflux conditions and then cooled to room temperature. The crude product was filtered, dried, and crystallized from ethanol to obtain pure compound 1c. Yield: 88%; mp 146 - 148°C. IR (KBr); ν cm^-1^: 2938, 1660. ^1^HNMR (500 MHz, DMSO-d6) δ: 8.08 - 8.41 (m, 1H), 7.64 - 7.77 (m, 1H), 7.44 - 7.55 (m, 1H), 7.32 - 7.40 (m, 1H), 3.60 - 4.54 (m, 7H, 2CH_2_ and NCH_3_). Mass; m/z (%): 201.2 (18), 181.2 (55), 176.2 (83), 159.2 (78), 151.1 (100), 138.1 (15), 131.1 (34), 116.1 (12), 111.1 (22), 104.1 (23), 89.1 (13), 75.1 (21), 64.1 (9).

##### 3.1.1.2. Ethyl 1-(2-(1-Methyl-1H-indazole-3-carboxamido)ethyl) piperidine-4-carboxylate (1d)

A mixture of 2-(1-methyl-1H-indazol-3-yl)-4,5-dihydrooxazole 1c (2 mmol), ethyl piperidine-4-carboxylate (2.1 mmol), and p-toluenesulfonic acid (2.1 mmol) was refluxed in dry xylene for 48 hours. Water (25 mL) was added and mixed well. The xylene layer was separated, and anhydrous sodium sulfate was added. After filtration, the solvent was completely removed by rotary evaporator, and the resulting oil was purified by flash chromatography (EtOAc: Hexane; 10:90). The purified solid was crystallized in ethanol to obtain pure compound 1d. Yield: 73%; mp 145-147°C. IR (KBr); ν cm^-1^: 2955, 1705, 1664. ^1^HNMR (500 MHz, DMSO-d6) δ: 8.30 (d, J = 7.8 Hz, 1H), 8.18 (d, J = 7.8 Hz, 1H), 8.05 (s, 1H, NH), 7.35 - 7.55 (m, 2H), 4.69 (t, J = 7.7 Hz, 2H, OCH2), 4.10 - 4.24 (m, 6H), 4.04 (s, 3H, NCH_3_), 3.05 - 3.55 (m, 1H), 2.50 - 2.82 (m, 2H), 1.50 - 1.82 (m, 2H), 1.25 (t, J = 7.7 Hz, 3H, CH_3_). ^13^CNMR (125 MHz, DMSO-d6): 175.32, 162.81, 162.56, 141.15, 136.89, 134.33, 126.80, 123.65, 122.31, 109.32, 63.96, 63.90, 45.02, 41.17, 38.28, 36.01, 28.59, 27.35, 14.18. Mass; m/z (%): 359.3 (45), 314.3 (55), 170.2 (100), 142.2 (6), 157.2 (8), 142.2 (6), 124.4 (5), 106.4 (6), 77.1 (5).

##### 3.1.1.3. Ethyl 1-(1-Methyl-1H-indazole-3-carbonyl) piperidine-4-carboxylate (2a)

A mixture of 1-methyl-1H-indazole-3-carbonyl chloride 1a (2 mmol), ethyl piperidine-4-carboxylate (2.1 mmol), and potassium carbonate (2.5 mmol) was stirred in dry acetonitrile overnight at room temperature. After evaporation of the solvent, the solid material was separated and dried at room temperature. Crystallization was carried out in ethanol to obtain pure compound 2a. Yield: 83%; mp 165 - 167°C. IR (KBr); ν cm^-1^: 2955, 1724, 1664. ^1^HNMR (500 MHz, CDCl3) δ: 8.39 (d, J = 7.5 Hz, 1H), 8.23 (d, J = 7.5 Hz, 1H), 7.25 - 7.55 (m, 2H), 4.15 - 4.32 (m, 2H), 4.04 (s, 3H, NCH_3_), 4.00 (q, J = 7.5 Hz, 2H, OCH_2_), 3.11 - 3.65 (m, 1H), 2.50 - 2.82 (m, 2H), 1.50 - 1.82 (m, 2H), 1.27 (t, J = 7.5 Hz, 3H, CH3). ^13^CNMR (125 MHz, CDCl_3_): 174.32, 162.80, 162.79, 141.01, 136.81, 126.96, 123.63, 122.23, 109.50, 63.96, 45.02, 41.17, 38.28, 36.01, 28.59, 27.35, 14.18. Mass; m/z (%): 315.1 (35), 270.1 (9), 156.1 (100), 131.1 (16), 82.1 (65).

##### 3.1.1.4. 1-(1-Methyl-1H-indazole-3-carbonyl) piperidine-4-carboxylic acid (2b)

A mixture of compound 2a (2 mmol) was dissolved in 20 mL of ethanol, and 20 mL of sodium hydroxide solution (2N) was added. The mixture was heated at 60°C for 4 hours. The progress of the reaction was monitored by TLC. The pH of the solution was neutralized with 2N hydrochloric acid solution, and the volume of the mixture was reduced to half of the initial level. The pH of the mixture was adjusted to about 3, and extraction was carried out with ethyl acetate. After evaporation of the organic phase, purification was performed by flash chromatography using hexane:ethyl acetate (3:1) as the solvent. Yield: 72%; mp 188 - 191°C. IR (KBr); ν cm^-1^: 3225, 2955, 1714, 1664, 1580. ^1^HNMR (500 MHz, CDCl_3_) δ: 12.36 (bs, 1H, COOH), 7.95 (dd, J = 7.6 and J = 1.5 Hz, 1H), 7.69 (dd, J = 7.6 Hz and J = 1.8 Hz, 1H), 7.40 - 7.50 (m, 1H), 7.20 - 7.29 (m, 1H), 4.46 - 4.55 (m, 2H), 4.14 (s, 3H, NCH_3_), 3.01 - 3.05 (m, 1H), 1.81 - 1.95 (m, 2H), 1.55 - 1.59 (m, 2H). ^13^CNMR (125 MHz, CDCl_3_): 176.04, 162.09, 141.51, 137.91, 126.99, 123.77, 122.08, 121.96, 110.55, 51.69, 45.77, 41.70, 40.73, 28.39, 25.68. Mass; m/z (%): 287.1 (25), 159.1 (65), 142.1 (57), 128.2 (100), 82.1 (17).

##### 3.1.1.5. Ethyl (4-Chlorobenzoyl) glycinate (3b)

Compound 3b was prepared as reported in the literature (CAS No.: 39735-52-9). Yield: 72%; mp 95 - 97°C.

##### 3.1.1.6. N-(2-(Benzylamino)-2-oxoethyl)-4-chlorobenzamide (3c)

A mixture of 3b (2 mmol) and AlCl_3_ (2 mmol) was stirred in dry acetonitrile (30 mL) for 30 minutes, and then a mixture of triethylamine (2 mmol) and benzylamine (2 mmol) was added and stirred at room temperature overnight. The solid formed was filtered and washed with water. Crystallization was carried out in ethanol to obtain pure compound 3c. Yield: 85%; mp 135 - 137°C. IR (KBr); ν cm^-1^: 3305, 3276, 2938, 1644, 1596, 1544. ^1^HNMR (500 MHz, CDCl_3_) δ: 8.88 (t, J = 7.5 Hz, 1H, NH), 8.45 (t, J = 5.5 Hz, 1H, NH), 7.91 (d, J = 7.5 Hz, 2H), 7.55 (d, J = 7.5 Hz, 2H), 7.17 - 7.35 (m, 5H), 4.30 (d, J = 5.5 Hz, 2H, -NH-CH_2_-CO-NH-), 3.91 (d, J = 7.5 Hz, 2H, -NH-CH_2_-Ph). ^13^CNMR (125 MHz, DMSO-d6): 169.3, 165.95, 139.89, 133.28, 129.79, 128.78, 128.66, 127.61, 127.16, 43.22, 42.80. Mass; m/z (%): 304.1 (4), 302.1 (12), 170.1 (23), 168.1 (69), 139.1 (100), 106.1 (78), 91.1 (45), 75.1 (18), 51.6 (6).

##### 3.1.1.7. 2-(4-Chlorophenyl)-4,5-dihydrooxazole (4a)

A mixture of 4-chlorobenzoyl chloride 3a (2 mmol), bromoethylamine (2.1 mmol), and potassium carbonate (2.5 mmol) was refluxed in dry acetonitrile overnight. After evaporation of the solvent, the solid material was separated and dried at room temperature. Crystallization was carried out in ethanol to obtain pure compound 4a. Yield: 90%; mp 144 - 146°C. ^1^HNMR (500 MHz, DMSO-d6) δ: 7.85 (d, J = 7.8 Hz, 2H), 7.52 (d, J = 7.8 Hz, 2H), 4.40 (t, J = 7.5 Hz, 2H), 3.95 (t, J = 7.5 Hz, 2H). ^13^CNMR (125 MHz, DMSO-d6): 162.53, 136.58, 129.93, 129.19, 126.81, 68.05, 54.95. Mass; m/z (%): 181.1 (56), 151.1 (100), 139.1 (15), 125.1 (12), 111 (23), 102 (4), 89.1 (10).

##### 3.1.1.8. Ethyl 1-(2-(4-Chlorobenzamido)ethyl) piperidine-4-carboxylate (4b)

A mixture of 2-(4-chlorophenyl)-4,5-dihydrooxazole 4a (2 mmol), ethyl piperidine-4-carboxylate (2.1 mmol), and p-toluenesulfonic acid (2.1 mmol) was refluxed in dry xylene for 48 hours. Sodium hydroxide solution (2N, 30 mL) was added and mixed well. The organic layer was separated, dried, and anhydrous sodium sulfate was added. The residue was purified by flash chromatography using EtOAc: Hexane (1:4) as the mobile phase. The product was crystallized from ethanol to obtain pure compound 4b. Yield: 62%; mp 156 - 158°C. IR (KBr); ν cm^-1^: 1724, 1644. ^1^HNMR (500 MHz, DMSO-d6) δ: 8.46 (t, J = 7.0 Hz, 1H, NH), 7.85 (d, J = 7.5 Hz, 2H), 7.51 (d, J = 7.5 Hz, 2H), 4.06 (q, J = 7.5 Hz, 2H, -OCH_2_), 3.45 - 3.59 (m, 2H), 3.31 - 3.39 (m, 2H), 2.81 - 2.93 (m, 4H), 2.22 - 2.35 (m, 1H), 1.79 - 1.85 (m, 2H), 1.65 - 1.78 (m, 2H), 1.19 (t, J = 7.5 Hz, 3H, CH_3_). ^13^CNMR (125 MHz, DMSO-d6): 174.82, 165.52, 133.71, 129.55, 128.73, 60.25, 57.81, 40.64, 37.38, 28.37, 14.56. Mass; m/z (%): 340.1 (1), 338.2 (3), 295.1 (2), 293.1 (6), 185.1 (2), 183.2 (6), 170.1 (100), 139.1 (12).

##### 3.1.1.9. Benzyl 4-(4-Chlorobenzamido) butanoate (5)

A mixture of 4-chlorobenzoyl chloride 3a (2 mmol), benzyl 4-aminobutanoate (2.1 mmol), and potassium carbonate (2.5 mmol) was stirred in dry acetonitrile overnight at room temperature. The solvent was removed under vacuum, and 20 mL of water and 20 mL of EtOAc were added. The mixture was well mixed, and the organic phase was separated, dried, and filtered. The crude product was purified by column chromatography using EtOAc:hexane (1:5) as the mobile phase. The separated product was an oil. Yield: 62%. IR (KBr); ν cm^-1^: 1731, 1665. ^1^HNMR (500 MHz, DMSO-d6) δ: 8.56 (t, J = 7.0 Hz, 1H, NH), 7.87 (d, J = 7.5 Hz, 2H), 7.52 (d, J = 7.5 Hz, 2H), 7.33 - 7.56 (m, 5H), 5.08 (s, 2H, -OCH_2_), 3.21 - 3.39 (m, 2H), 2.43 (t, J = 7.2 Hz, 2H), 1.79 - 1.85 (m, 2H). ^13^CNMR (125 MHz, DMSO-d6): 174.29, 165.21, 136.95, 139.89, 133.23, 129.03, 128.33, 128.21, 127.87, 127.81, 127.36, 31.01, 24.38. Mass; m/z (%): 298.1 (10), 196.1 (12), 168.1 (16), 139.1 (25), 122.1 (65), 105.1 (100), 77.1 (12).

#### 3.1.1.10. Ethyl 1-(2-Oxo-2-(4-phenylpiperazin-1-yl)ethyl) piperidine-4-carboxylate (6b)

A mixture of 1-phenylpiperazine 6 (4 mmol), ethyl chloroacetyl chloride (4.1 mmol), and potassium carbonate (4.5 mmol) was stirred in dry acetonitrile (20 mL) at 60°C. The progress of the product was checked by TLC. The crude product (6a) was directly used for the next step. Ethyl piperidine-4-carboxylate (4.1 mmol), potassium carbonate (2.5 mmol), and 30 mL of DMF were added, and the mixture was refluxed in dry DMF for 6 hours. The solvent was evaporated under reduced pressure, and 20 mL of water and 30 mL of EtOAc were added. The organic phase was separated, dried on sodium sulfate, and filtered. The solvent was removed under vacuum, and the crude product was crystallized from ethanol to obtain pure compound 6b. Yield: 82%; mp 156 - 158°C. IR (KBr); ν cm^-1^: 1724, 1632. ^1^HNMR (500 MHz, DMSO-d6) δ: 7.22 (d, J = 7.5 Hz, 2H), 6.90 - 6.99 (m, 2H), 6.80 - 6.90 (m, 1H), 4.04 (q, J = 7.5 Hz, 2H, -OCH_2_), 3.69 (bs, 2H), 3.45 - 3.65 (m, 2H), 2.90 - 3.15 (m, 6H), 2.70 - 2.85 (m, 2H), 2.25 - 2.336 (m, 1H), 2.00 - 2.15 (m, 2H), 1.70 - 1.85 (m, 2H), 1.50 - 1.65 (m, 2H), 1.16 (t, J = 7.5 Hz, 3H, CH3). ^13^CNMR (125 MHz, DMSO-d6): 174.77, 167.96, 151.32, 129.43, 119.72, 116.27, 61.60, 60.25, 52.53, 49.46, 48.86, 45.48, 41.53, 28.49, 14.55. Mass; m/z (%): 359.1 (6), 314.3 (6.5), 170.2 (100), 142.2 (10), 106.2 (11), 77.1 (12), 44.1 (25).

### 3.2. Docking Methodology

For docking simulation studies and structure drawing, the Amber10 force field equation and Hamiltonian PM3, RHF were used, and the results were saved as PDB files (10, 11). The RCSB Protein Data Bank was utilized to find the crystal structures of GABA_A_ (6X3X) in complex with diazepam. Homology modeling was employed to compare the sequence of amino acids in all breaks and imperfections of the crystal structure (12). In the loop and terminal parts, missing amino acids were added, partial charges were fixed, and the MMFF94X force field equation was used to minimize the energy of the structure (13). Autodock 4.2 software was used for optimizing the receptor and ligands in the molecular docking study. To favor the conformation in the active site, induced fit and genetic algorithms were used (14). The calculation of hydrogen bonds and entropy changes was carried out using the MMFF94X force field equation for the best conformations. Validation was studied using the molecular conformation of the co-crystallized ligand. The results were analyzed using Discovery Studio 4.5 and MOE software (15).

### 3.3. In Vivo Anticonvulsant Activity

The supplier for the adult Charles Foster rats (male, average weight 150 ± 10 g) was the animal house of the Faculty of Pharmacy, TUMS. A package included 9 groups of 6 rats housed in normal cages (16, 17). All animals had free access to food and water during the experiment. The experiments were conducted between 9 AM and 2 PM to prevent the negative effects of environmental changes. All experiments were performed in accordance with the standards and pharmacological guidelines for the conservation of experimental animals (18, 19) For all experiments, the rats were transferred to the lab 60 minutes before starting the tests to acclimate to the lab environment. Animals were randomly divided into experimental groups, and each rat was used only once for the experiments.

In this research, diazepam and the synthesized compounds were administered via intraperitoneal injection (i.p.) at a dose of 10 mg/kg^-1^ of rat body weight. Diazepam and other compounds were dissolved in a solution containing 10% DMSO in distilled water. PTZ (50 mg/kg^-1^) was injected i.p. to induce tonic-clonic generalized convulsions. The dosages of the synthesized derivatives (10 mg/kg^-1^) and PTZ (50 mg/kg^-1^) were selected based on prior studies (15). In experiments with continuous treatments, the interval between the administration of diazepam or the synthesized derivatives and PTZ was 30 minutes (17).

To evaluate the antiseizure effects of the test compounds and diazepam, each group containing 6 rats was chosen. Initially, the test compounds were dissolved in 10% aqueous DMSO, and then 10 mg/kg^-1^ of each compound was administered intraperitoneally 30 minutes prior to the induction of tonic-clonic convulsions by 50 mg/kg^-1^ PTZ solution. The latency of tonic-clonic convulsions was recorded for 60 minutes. To evaluate the possible effect of the vehicle, a similar process was performed for 10% aqueous DMSO as the control compound and diazepam as the standard drug.

### 3.4. Statistical Analysis

**Figure 2.**
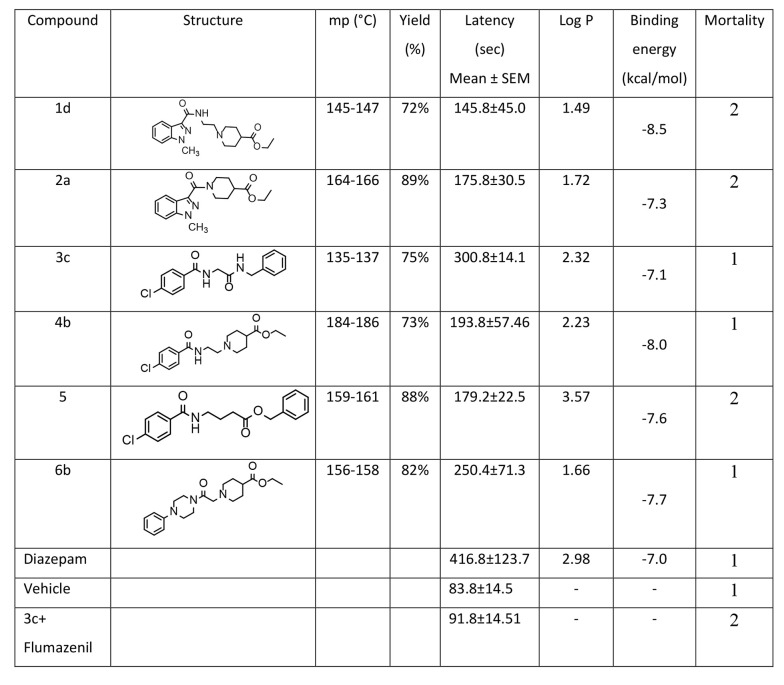
Structures, physicochemical data, log p, docking binding energies of the test compounds and their biological properties

In this research, statistical analysis was conducted using SPSS (version 25, 2017, USA). The data and results of convulsion thresholds are presented as the mean ± SEM of tonic-clonic convulsion latency in each trial group. The results were analyzed by one-way analysis of variance (ANOVA) with Tukey’s post hoc test for multiple comparisons between groups. In all experiments, a probability value of less than 0.05 was considered statistically significant. Pharmacological effects and anticonvulsant parameters of the synthesized derivatives, including antiseizure activity (latency) and mortality, are demonstrated in Figure 2. 

## 4. Results and Discussion

### 4.1. Chemistry

**Figure 3.**
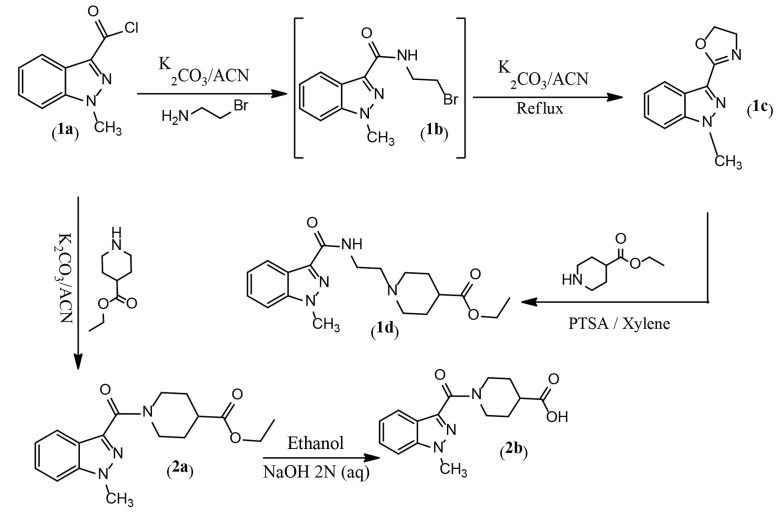
Synthesis routes of compounds 1d, 2a and 2b

The synthesis of designed derivatives 1d and 2b was performed through amidation, cyclization, and nucleophilic substitution reactions as described in Figure 6. In this study, compound 1c was first synthesized from a reaction between 1-methyl-1H-indazole-3-carbonyl chloride 1a and bromoethylamine in dry acetonitrile. Our experiments showed that the reaction involved the formation of intermediate 1b, and a mixture of 1b and 1c was formed at room temperature. Therefore, the solution was refluxed to yield 1c as the main product. Subsequently, ethyl 1-(2-(1-methyl-1H-indazole-3-carboxamido)ethyl)piperidine-4-carboxylate 1d was synthesized from a reaction between 2-(1-methyl-1H-indazol-3-yl)-4,5-dihydrooxazole 1c and ethyl piperidine-4-carboxylate in xylene as a solvent, with p-toluenesulfonic acid (PTSA) as a catalytic reagent at 150°C. In the second pathway, ethyl 1-(1-methyl-1H-indazole-3-carbonyl)piperidine-4-carboxylate 2a was prepared from a reaction between 1-methyl-1H-indazole-3-carbonyl chloride 1a and ethyl piperidine-4-carboxylate in dry acetonitrile with potassium carbonate at room temperature (Figure 3). The alkaline hydrolysis of 2a yielded the related carboxylic acid.

**Figure 4.**
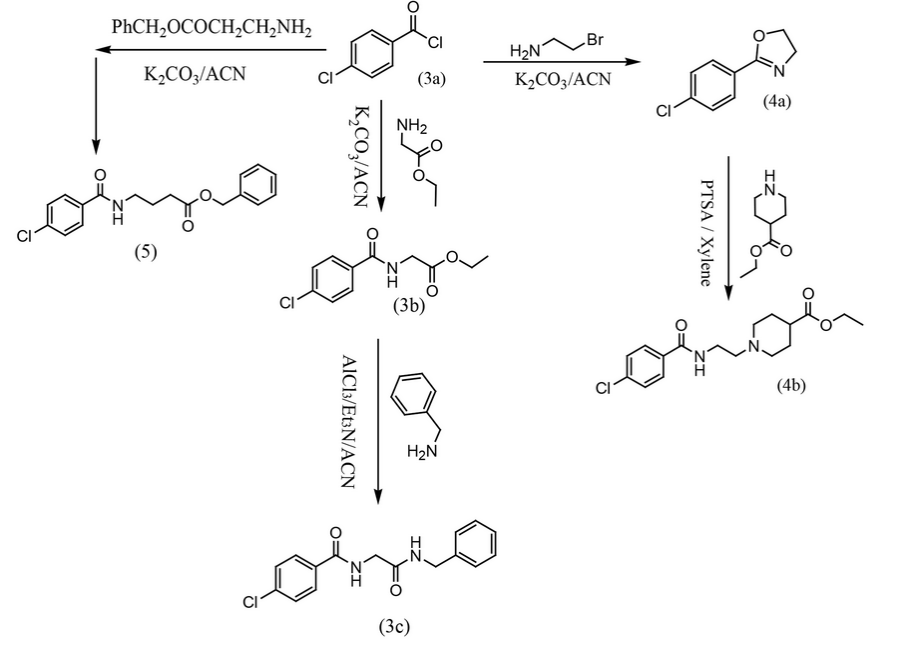
Synthesis routes of compounds 3c, 4b and 5

In the second section, ethyl (4-chlorobenzoyl)glycinate 3b was prepared from the reaction of 4-chlorobenzoyl chloride 3a and glycine ethyl ester with potassium carbonate in dry acetonitrile at room temperature. Then, N-ethyl (4-chlorobenzoyl)glycinate 3b was stirred with AlCl3 for 30 minutes in dry acetonitrile. A mixture of triethylamine and benzylamine was added to the previous solution, and the mixture was stirred at room temperature overnight to prepare N-(2-(benzylamino)-2-oxoethyl)-4-chlorobenzamide 3c. In another route, 2-(4-chlorophenyl)-4,5-dihydrooxazole (4a) was synthesized from a reaction between 4-chlorobenzoyl chloride (3a) and bromoethylamine in dry acetonitrile with potassium carbonate under reflux conditions. In the final step, ethyl 1-(2-(4-chlorobenzamido)ethyl)piperidine-4-carboxylate (4b) was synthesized from a reaction between 2-(4-chlorophenyl)-4,5-dihydrooxazole (4a) and ethyl piperidine-4-carboxylate in dry xylene as a solvent, with p-toluenesulfonic acid (PTSA) as a catalytic reagent at 150°C. In the third pathway, benzyl 4-(4-chlorobenzamido)butanoate (5) was synthesized from a reaction between 4-chlorobenzoyl chloride (3a) and benzyl 4-aminobutanoate in dry acetonitrile with potassium carbonate at room temperature (Figure 4). 

**Figure 5.**
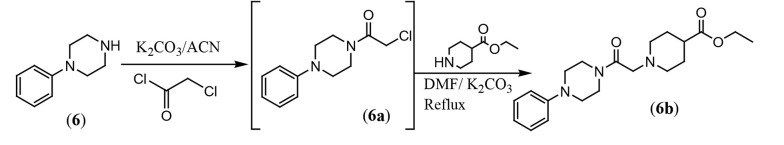
Synthesis routes of compound 6b

In the third section, 2-chloro-1-(4-phenylpiperazin-1-yl)ethan-1-one (6a) was synthesized from a reaction between 1-phenylpiperazine (6) and chloroacetyl chloride in dry acetonitrile with potassium carbonate at 100°C. Compound 6a was then directly used for the preparation of ethyl 1-(2-oxo-2-(4-phenylpiperazin-1-yl)ethyl)piperidine-4-carboxylate (6b) through a reaction between 6a and ethyl piperidine-4-carboxylate in dry DMF with potassium carbonate at 160°C (Figure 5). The yields of the final compounds ranged from 75% to 85%. The structures of the final compounds were confirmed using IR, mass, and NMR spectra.

### 4.2. Pharmacology

**Figure 6.**
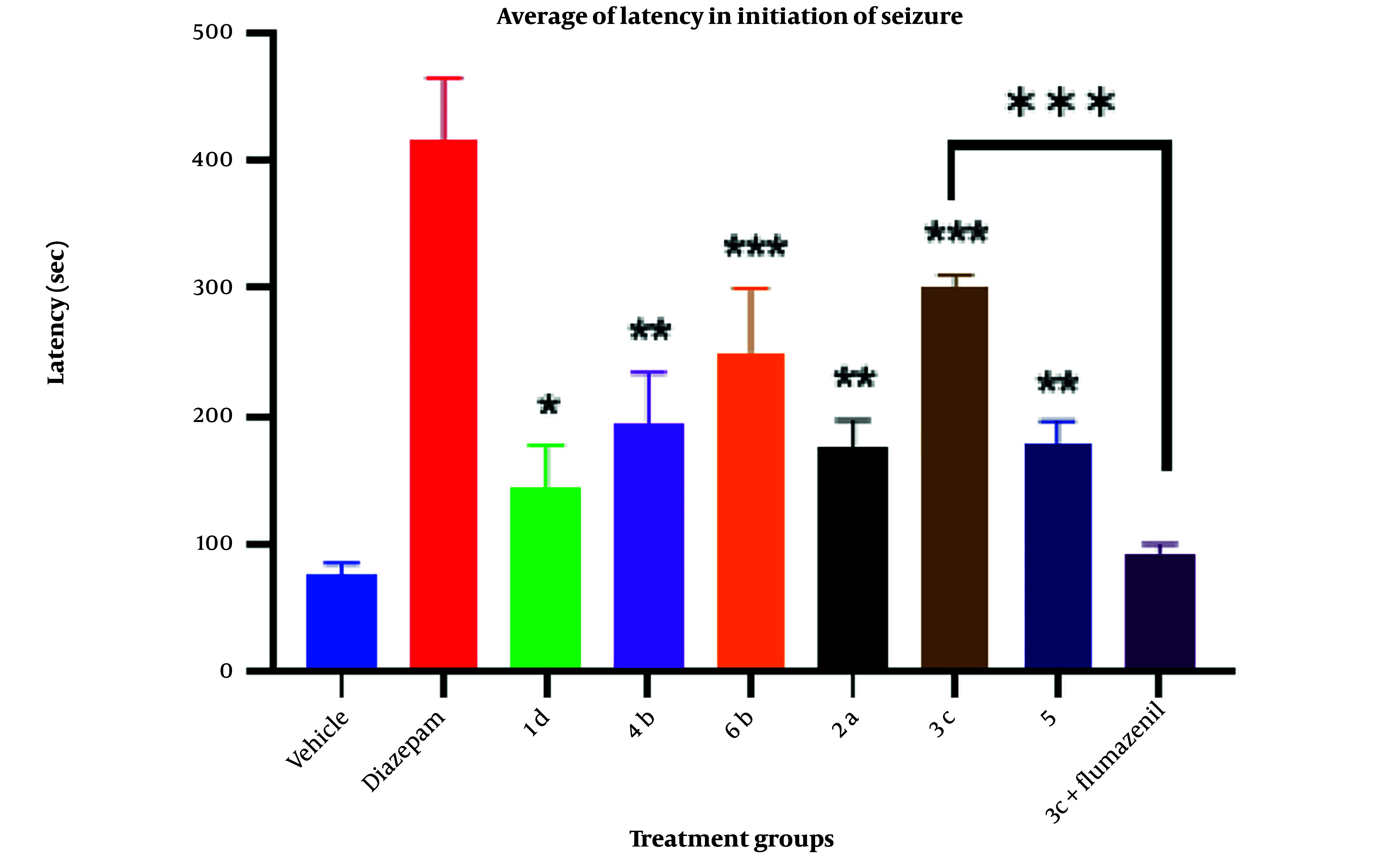
Effects of intraperitoneal administration of compound 1d, 2a, 3c, 4b, 5, and 6b (10 mg mL-1) on the latency after using pentylenetetrazole (PTZ) in rat in comparisons with diazepam control groups. Animal received test compounds 30 min before PTZ administration. The results are presented as mean ± SEM. The following symbol were used for interpretation of statically aspects: * P < 0.05, ** P < 0.01, *** P < 0.001 compared to vehicle and diazepam.

The anticonvulsant activities of the synthesized derivatives, including antiseizure effect, log P, and mortality rate, are illustrated in Figure 2. Additionally, the activity of the test compounds was compared with DMSO (carrier) as the control group and diazepam as the standard drug on latency after using PTZ as intraperitoneal administration. Although all the synthesized compounds showed less activity compared to diazepam in reversing the effects of PTZ, the outcomes of the one-way ANOVA test demonstrated that derivative 3c has a greater protective effect than DMSO (P < 0.05) against PTZ-induced convulsions. Furthermore, compounds 3c and 6b showed significantly more protective effects than the control, with a P-value of less than 0.001. Conversely, compounds 4b, 2a, and 5 demonstrated similar results with a P-value of less than 0.01. Compound 3c, as the most potent compound, was selected to investigate whether the inhibition of chloride ion channels in the GABAergic pathway could be considered the mechanism of its anticonvulsive property using the flumazenil test. The effects of acute intraperitoneal injection of flumazenil, a GABA_A_ receptor antagonist, on PTZ-induced CST were evaluated (Figure 6). Flumazenil, at an ineffective dose of 0.25 mg/kg^-1^, was injected 20 minutes prior to the injection of 3c. Compound 3c showed a significant reduction in its anticonvulsant activity after pretreatment with a low dose of flumazenil, indicating that the GABAergic system could be considered an important route in the antiseizure activity of 3c.

Attention to the structure of compounds in Figure 2 shows that all of them share a similar property: An aromatic ring connected to the negative part of the molecule by different spacers. In all cases, the spacers contain two carbonyl groups, either amide-ester or amide-amide linkers. However, despite the noticeable differences in log P and binding energy between 1d and 4b, the latency time did not show any significant discrepancy. Similar behavior was observed among others (1a, 2a, and 5). The only exception among the test series compounds is 3c, which showed a more significant latency time compared to the others. Interestingly, compound 3c lacks a potentially real negative charge; instead, it contains a partially negative charge provided by the amide group. Compound 1d, as the weakest compound, includes an N-methyl indazole ring as the aromatic core with a potential real negative charge but did not show remarkable biological activity, despite 1d exhibiting considerable negative binding energy in docking studies.

As a superficial conclusion, it can be mentioned that the indazole ring is not a good candidate for anticonvulsant activity compared to the 4-chlorophenyl ring. Finally, compound 3c could be introduced as a new lead for developing drug discovery in the area of epilepsy in future research.

### 4.3. Molecular Docking Study

**Figure 7.**
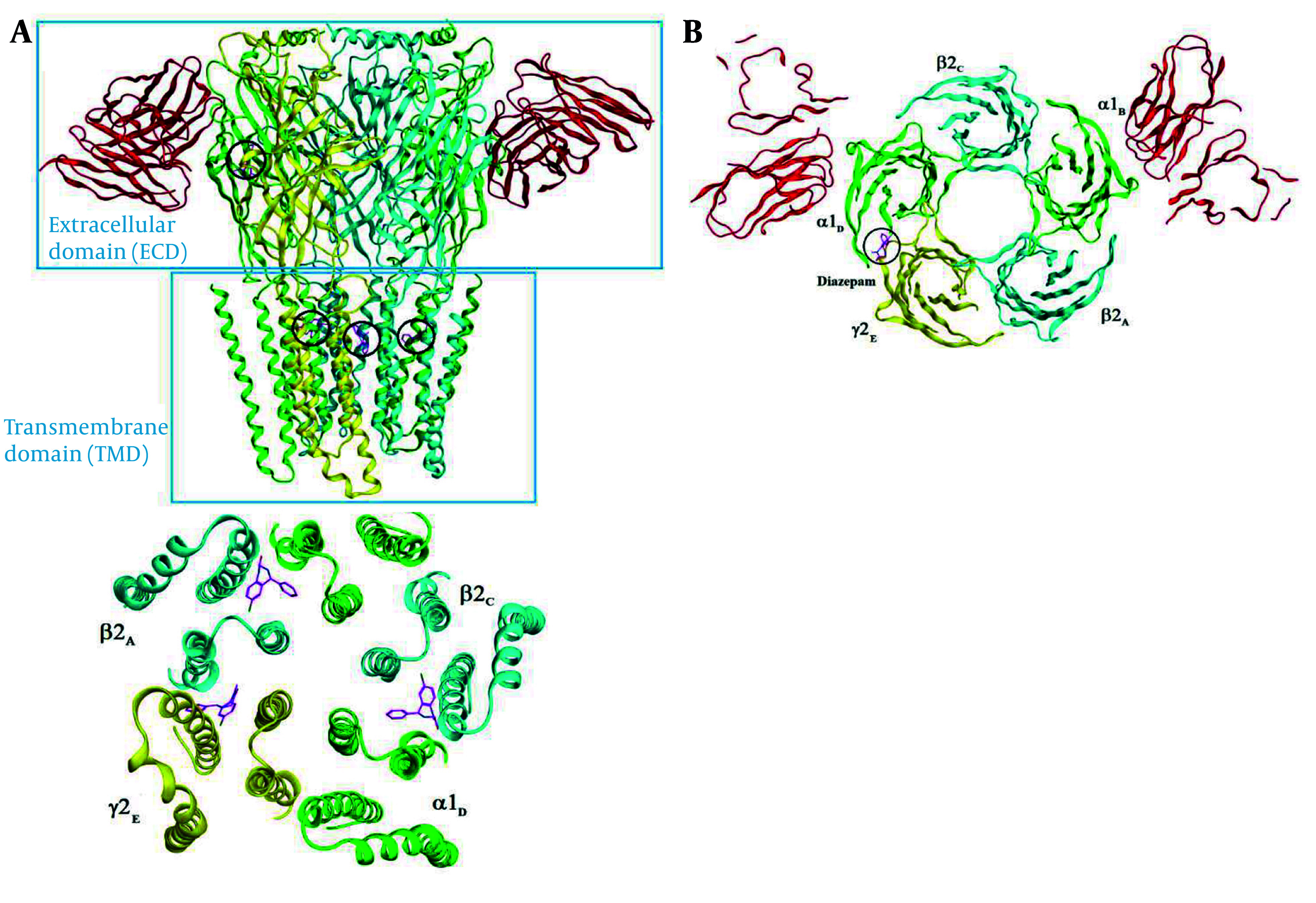
Representation of extracellular and trans membrane domains of GABA_A_ receptor and diazepam binding sites (black circle illustrated the position of diazepam binding site)

A molecular modeling study was performed to investigate the binding interactions between all synthesized compounds at the allosteric site of benzodiazepine (BNZ). All synthesized compounds were positioned correctly, and their binding energies were comparable with the co-crystallized compound. The 3D conformation of the compounds and the ligand-protein schematic interactions are shown in Figure 7. The best score is related to compound 1d, which interacts with the amino acids of the active site, showing similarity with diazepam. The key amino acids of the binding pocket are SER 205 and SER 206, which interact through hydrogen bonding in both molecules. Moreover, PHE 77, TYR 58, TYR 210, PHE 100, and TYR 160 participate in similar interactions with both molecules.

**Figure 8.**
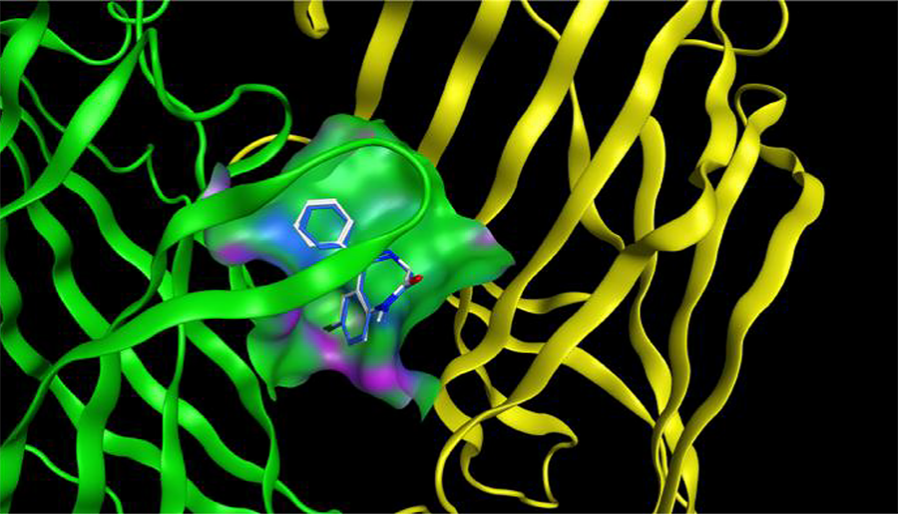
Validation of docking protocol using crystallized ligand (diazepam) conformation: An RMSD assessment

The docking protocol was validated by re-docking the crystallized ligand (diazepam) into the active site of the target protein. The root-mean-square deviation (RMSD) of 1.43 Å between the crystallized and docked ligand conformations indicates the reliability of the docking approach for reproducing experimental binding poses (Figure 8). 

**Figure 9.**
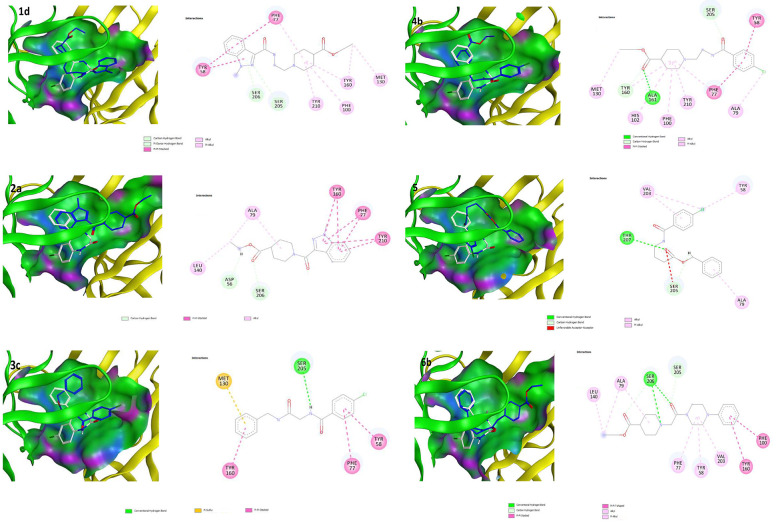
Superposition of the synthesized compounds on allosteric binding site [diazepam (white) and synthesized compounds (blue)] and corresponding 2D graphical presentation of each ligand interaction with binding pocket’s amino acids

Most anticonvulsant drugs generally contain a heterocyclic aliphatic or aromatic ring with at least one nitrogen atom (16). Among anticonvulsant drugs, tiagabine, which features a piperidine ring, has been considered for the design of new types of anticonvulsant drugs due to its biological effects. In this research, a piperidine ring or GABA-like linker was used to connect an aryl system with a carboxylic ester group. Diazepam and BNZs are an important class of anticonvulsant drugs. The main structural sections for their activity include lipophilic aryl rings, hydrogen bonding moieties, and electron-accepting groups (17, 18). Additionally, there are four principal parameters for antiseizure drugs: Lipophilic Scale, aryl rings (lipophilic moiety) as π-π charge transfer, (–CONH moiety) as hydrogen donor and acceptor, and (C=N moiety) as an electron donor and π-π charge transfer (19, 20). The size of aryl rings and the nature of substituents on aryl rings affect pharmacokinetic and pharmacodynamic properties. To increase the likelihood of CNS influence, the ester form of carboxylic acid was used as a prodrug in an in vivo animal model. Among them, compound 3c, as the most potent compound, showed a log P of 2.32, indicating sufficient capability for CNS penetration. Most of our synthesized derivatives exhibit antiseizure activities with a good correlation between experimental results and molecular modeling data. Compound 3c has the most anticonvulsant effects and the least mortality, making it a target for subsequent drug design and biological studies. On the other hand, antagonization of the BNZ receptor by flumazenil partially reduced the anticonvulsant activity of compound 3c on tonic-clonic PTZ-induced seizures (Figure 9), demonstrating that the GABA_A_ receptor, as well as sodium channels, participates in the observed effects.

## 5. Conclusions

The current study presents a new set of compounds with potential anticonvulsant activity. Despite the fact all the synthesized compounds have weaker effects compared to diazepam in PTZ-in vivo model, they significantly prolonged the latency time compared to the control. Study of mechanism of action for compound 3c showed the GABAergic system is involved in this biological effects. Compound 3c could be considered as a new probe for developing of drug discovery in future study. 

## Data Availability

The data presented in this study are provided in the main text and are openly available for readers upon request.
